# Gegen huangqin huanglian decoction for children rotavirus enteritis: A protocol for systematic review and meta-analysis

**DOI:** 10.1097/MD.0000000000023376

**Published:** 2020-12-04

**Authors:** Yifang Wu, Xiang Tu, Xiao Liang, Jing Chen, Xuemei Wan, Tianhong Zhang, Jingrong Jiang, Sen Zhong

**Affiliations:** Hospital of Chengdu University of Traditional Chinese Medicine, Chengdu, Sichuan Province, China.

**Keywords:** Gegen Huangqin Huanglian Decoction, protocol, rotavirus enteritis, systematic review

## Abstract

**Background:**

Rotavirus infection is the main cause of severe dehydration enteritis in children under 5 years old. It gives rise to malnutrition and even death in children even though there were rotavirus vaccines. However, there is no effective anti-virus drugs for rotavirus, supporting treatments are used in the clinics. Traditional Chinese medicine (TCM) has been treating diarrhea for many years. Gegen Huangqin Huanglian Decoction (GHHD)is a classic prescription for diarrhea in TCM. With the development of clinical trials and basic studies, GHHD has been proved that a good curative effect on diarrhea. Therefore, a systematic review is necessary to improve available evidence for GHHD in therapy of children under 5 years old with rotavirus enteritis.

**Methods:**

Different studies from various databases will be involved in this study. Only randomized controlled trials of rotavirus enteritis patients diagnosed with Guidelines for the Treatment of Acute Gastroenteritis in Outpatient Pediatrics, which released by the Washington International Children's Medical Center, Zhu Futang's Practical Pediatrics (7 th Edition), and the 2016 clinical practice guidelines for children with acute infectious diarrhea in China. We will search the literature in the databases from China Conference Paper Database, manual searching. Electronic database includes PubMed, Embase, Cochrane Library, Web of Science, CNKI (China National Knowledge Internet), WanFang, VIP (Chongqing VIP), and CBM (China Biomedical Literature CDROM Database). The primary outcomes include the total effective rate, the time of stopping diarrhea, the level of IL-6 serum concentration, fecal microflora ratio, the conversion of fecal rotavirus antigen. The secondary outcomes include clinical efficacy and the quantitative integral of TCM symptom, recovery time of stool character, treatment period. Besides, incidence of adverse events (such as irritation and toxicity) and costs will be also considered. Data will be extracted by 2 researchers independently, risk of bias of the meta-analysis will be evaluated based on the Cochrane Handbook for Systematic Reviews of Interventions. All data analysis will be conducted by data statistics software Review Manager V.5.3 and Stata V.12.0.

**Results:**

This study will synthesize and provide high-quality evidence based on the data of the currently published GHHD for the treatment of children rotavirus enteritis, in terms of the total effective rate, the time of stopping diarrhea, the level of IL-6 serum concentration, fecal microflora ratio, stool rotavirus antigen, clinical efficacy and the quantitative integral of TCM symptom, recovery time of stool character, treatment period, and safety.

**Conclusion:**

This systematic review aims to evaluated the benefits and harms of GHHD for the treatment of children rotavirus enteritis reported in randomized controlled trials, and provide more options for clinicians and patients to treat children rotavirus enteritis.

**Registration number:**

INPLASY2020100023

## 1 Introduction

### 1.1 Description of the condition

Diarrheal disease was the fourth leading cause of death among children younger than 5 years old in 2015.^[1]^ Furthermore, rotaviruses are the most common cause of severe gastroenteritis worldwide and of diarrhea deaths in developing countries.^[2,3]^ Several studies have shown rotavirus and enteric adenovirus are the most frequent causes of nonbacterial gastroenteritis, particularly in children in the age range of 0 to 5 years.^[4]^ Rotavirus can be infected several times in children’ lives, and almost every child is infected up to the age of 5.^[5]^

Although rotavirus vaccination was started more than a decade ago, rotavirus infection still causes more than 200,000 deaths a year. In 2003, 114 million cases of rotavirus infection in children under the age of 5were reported worldwide, of which 24 million need outpatient treatment and 2.3 million needed hospitalization.^[6,7]^ In 2013, rotavirus were associated with an estimated >200,000 fatalities in children < 5 years of age globally, and > 90% of children with fatal rotavirus infections live in low-income countries.^[8]^ Rotavirus is 1of the 13 diarrheal etiologic agents measured in the Global Burden of Disease Study 2016 (GBD 2016).^[1,8]^ This study describes the incidence and mortality of rotavirus infection among children younger than 5 years and reveals the urgent need for interventions to reduce diarrhea risk, treat severe diarrhea episodes, and prevent rotavirus diarrhea. It is estimated that 128500 (95% uncertainty interval (UI), 104500-155600) children under the age of 5 died of rotavirus infection worldwide in 2016. Rotavirus infection caused more than 258 million (95% uncertainty interval (UI), 193 million – 341 million) diarrhea episodes in children under the age of 5 in 2016.^[9]^

In 2016, rotavirus was the third leading cause of mortality in children under 5 years old, after malaria and streptococcus pneumoniae.^[8]^

### 1.2 Description of the intervention and how it might work

There is no effective drug for rotavirus infection at present. Routine treatment for children rotavirus enteritis including fluid supplement therapy, dietary therapy, zinc supplementation, drug therapy including probiotics replacement therapy, montmorillonite, antibiotics, Traditional Chinese Medicine (TCM), and so on. The formula of Gengen Huangqin Huanglian Decoction (GHHD), first recorded in the book of *shanghan zabing lun* written by Zhang Zhongjing, is composed of *Puerariae Lobatae Radix (Gegen), Scutellariae Radix (Huangqin), Coptidis Rhizoma (Huanglian)*, *glycyrrhiza (Gancao)*. It is recorded in the book that GHHD can be used to treat diarrhea, vomiting and fever caused by exterior pathogen. The above symptoms are also the main symptoms of rotavirus enteritis. The main pathogenesis of children rotavirus enteritis is dampness-heat in stomach and intestines mostly.^[10–12]^ GHHD could clear away the heat and dry dampness. Therefore, GHHD has been widely used in the clinical treatment of children rotavirus enteritis and achieve good results.^[13–15]^

In addition, pharmacological studies have shown that after treatment with GHHD, the level of serum IL-1, IL-2 and IL-6 were decreased, symptoms and sigs disappeared, and pathological changes were basically restored.^[16]^ In vitro Gegen Huangqin Huanglian micro-pellet can not only prevent human rotavirus invasion of host cells, but also can inhibit rotavirus in host cells with in the biological synthesis of the process.^[17]^ Berberine, the main component of *Coptidis Rhizoma (Huanglian)*, can inhibit both acute and chronic inflammation. One study showed that berberine inhibited COX-2 expression and prostaglandin E2 (PGE2) levels.^[18]^

### 1.3 Objectives

This review aims to systematically evaluate the benefits and harms of GHHD for children rotavirus enteritis patients reported in randomized clinical trials (RCTs). We look forward to provide more reliable evidence on as a supplementary treatment, dose GHHD really improve the efficacy of treatment on children rotavirus enteritis.

## 2 Methods

### 2.1 Protocol registration

The protocol of the systematic review has been registered in https://inplasy.com/inplasy-2020-10-0023/, and the registration number is INPLASY2020100023. This systematic review protocol will be conducted and reported strictly according to the Preferred Reporting Items for Systematic Reviews and Meta-Analyses Guidelines for Protocols (PRISMA-P).^[19]^ And the important protocol amendments will be documented in the full review.

### 2.2 Inclusion criteria

#### 2.2.1 Study design

Only clinical RCTs (expect Quasi-RCTs and cluster RCTs) will be selected published in both Chinese and English. Animal mechanism studies and non-RCTs will be excluded. Article that substantially overlaps with another published article in print or electronic media will be excluded. Duplicate publications produced by a single experiment and published as separate papers with different criteria for measuring results, priority will be given to original publications and other publications will be excluded. The language and time of publication will not be restricted.

#### 2.2.2 Participants

The patients of children under the age of 5 with rotavirus enteritis (using criteria from *Guidelines for the Treatment of Acute Gastroenteritis in Outpatient Pediatrics,* which released by the Washington International Children's Medical Center, *Zhu Futang*'*s Practical Pediatrics (7 th Edition),* and the 2016 clinical practice guidelines for children with acute infectious diarrhea in China.^[20,21]^) These patients will not be included: patients with acute complications of rotavirus enteritis, like severe dehydration or serious electrolyte disorder and consciousness disorder; patients with immune deficiency, severe cardiovascular, liver and kidney, hematopoietic system or other primary diseases, mental illness; patients with allergic history and hypersensitivity to relevant drugs.

#### 2.2.3 Interventions

Both groups were cured with conventional children rotavirus enteritis treatments recommended by the 2016 Clinical Practice Guidelines for Children with Acute Infectious Diarrhea in China.

Including fluid replenishment therapy to maintain balance of water and electrolyte, diet therapy, drug therapies (such as probiotics, montmorillonite, zinc supplementation, etc^[22]^) The experiment group used GHHD or modified GHHD, while the control group applied for placebo or no TCM treatment. In addition, the 2 groups did not take any drugs that interfered with the outcome indicators. The choice of routine treatment in each RCT does not have to be exactly consistent, but GHHD or modified GHHD should be the only difference between interventions and controls.

#### 2.2.4 Outcomes

The primary outcomes include the total effective rate (%), the time of stopping diarrhea (there were 2 times of defecation or no defecation for 24 hours), the level of IL-6 serum concentration, fecal microflora ratio, the conversion of fecal rotavirus antigen. The secondary outcomes include the quantitative integral grade of TCM symptoms,^[23]^ recovery time of normal stool, treatment period (from the first symptom appear to recovery). Besides, incidence of adverse events (such as irritation and toxicity) and costs will be also considered.

According to *the Diagnosis and Treatment of Diarrhea in China*, the standard of clinical effect is those, recovery: all symptoms and signs disappear or almost appear after 72 hours treatment, TCM symptom grade ≥ 95%. Markedly effective: the time and character of stool back to normal status after 72 hours treatment, TCM clinical symptoms and sign improved significantly, 70% ≤ TCM symptom grade < 95%. Effective: the time and character of stool improved after 72 hours treatment, TCM clinical symptoms and sign improved, 30% ≤ TCM symptom grade < 70%. Ineffective: the time and character of stool not improved or even worse after 72 hours treatment, TCM symptoms and signs are not improved or worsen, TCM symptom grade < 30%.

### 2.3 Search methods

#### 2.3.1 Search resources

This review will include grey literature sourced from China Conference Paper Database on the corresponding website, manual searching. Electronic database includes PubMed, EMBASE, Cochrane Library, Web of Science, CNKI, WanFang, VIP, and CBM will also be retrieved. We will simply present the search process of PubMed (Table 1). The data will be searched in English and Chinese databases from their inception to August 2020, adjusting different search methods according to different Chinese and English databases.

**Table 1:** PubMed search strategy.

Example of Cochrane search strategy
Number Search terms
MeSH descriptor: (rotavirus) explore all trees
((rotavirus enteritis ^∗^) or (rotavirus gastroenteritis ^∗^) or (infantile diarrhea ^∗^) or (children diarrhea ^∗^)): ti, ab, kw
OR 1-2
Gegen [All Fields] AND Huangqin [All Fields] AND Huanglian [All Fields] AND Decoction [All Fields]
Gegen [All Fields] AND Huangqin [All Fields] AND Huanglian [All Fields] AND Tang [All Fields]
Gegen Huangqin Huanglian Tang [All Fields] OR Gegen Qin Lian Decoction [All Fields]
4 OR 5OR 6
MeSH descriptor: (randomized controlled trials) explore all trees
((random ^∗^) OR (randomly ^∗^) OR (random allocation ^∗^) OR (placebo ^∗^) OR (single blind ^∗^) OR (double blind ^∗^) OR (clinical trials ^∗^) OR (randomized control trial ^∗^) OR (RCT ^∗^) OR (controlled clinical trials ^∗^)): ti, ab, kw
OR 8–9
3 AND 7 AND 10

#### 2.3.2 Search strategies

The following MeSH terms and their combinations will be searched:

GHHD OR Gegen Huangqin Huanglian Tang; OR Gegen Qin Lian Decoction; rotavirus enteritis OR rotavirus gastroenteritis OR rotavirus; and children OR infant OR child OR baby. Clinical trials.

### 2.4 Data collection and analysis

#### 2.4.1 Studies selection

**Figure 1:**
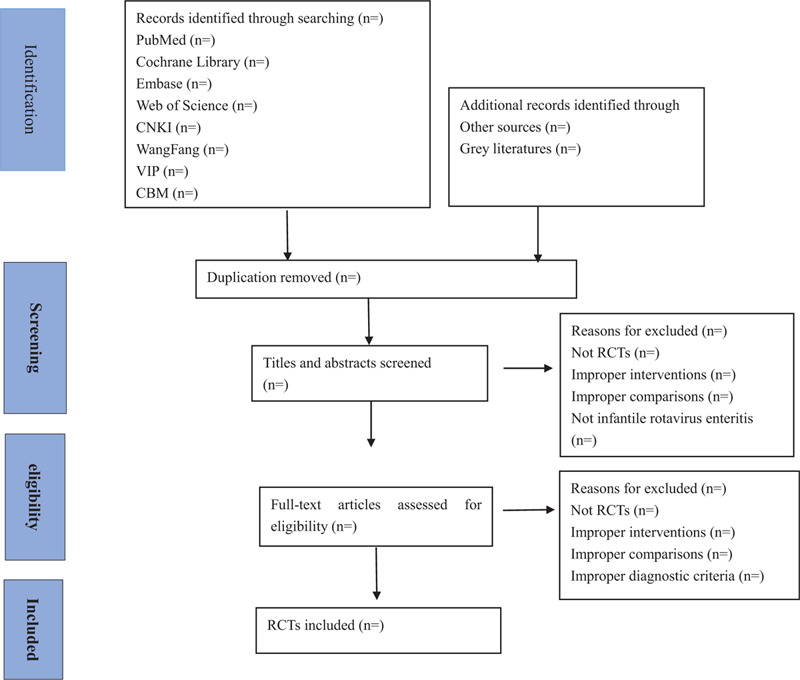
Flow chart of the study selection.

There will be 2 researchers carry out the literatures that meet the requirements independently using endnote X9 software. We will make the preliminary selection by screening titles and abstracts firstly. Secondly, we will download full text of the relevant studies and read carefully for further selection according to the inclusion criteria. If there is any different opinion, 2 researchers will discuss and reach an agreement. If a consensus could not be reached, there will be a third researcher who makes the final decision. Details of the selection process were shown in the flow chart (Fig. 1). Finally, the results were cross-checked repeatedly by reviewers.

#### 2.4.2 Data extraction

Two investigators will read all the eligibility of each included text and extract data by using a standardized template independently. The standardized template include the following information:

(1) basic information (title, year of publication, the first authors name, fund source),
(2) study method (design, blinding, sample size),
(3) information of participants (number of sample in the group, gender, age, course of disease, stool times in 24 hours, the quantitative integral grade of TCM symptoms),
(4) information of treatment (interventions and controls, medicine, dose, frequency, duration),
(5) outcomes: the primary outcomes include the total effective rate (%), the time of stopping diarrhea (there were 2 times of defecation or no defecation for 24 hours), the level of IL-6 serum concentration, fecal microflora ratio, the conversion of fecal rotavirus antigen. The secondary outcomes: the quantitative integral grade of TCM symptoms, recovery time of normal stool, treatment period (from the first symptom appear to recovery), incidence of adverse events and costs.
(6) *P*-value.

If we could not reach an agreement, a third researcher would make the final decision. One researcher would contact the corresponding author by telephone or e-mail for more information when the reported data were insufficient or ambiguous. The above information was finally cross-checked by 2 researchers.

#### 2.4.3 Assessment of risk of bias

The risk of bias (ROB) assessment tool which provided by the Cochrane Handbook for Systematic Review of Interventions would be used for the quality assessment of RCTs.^[24,25]^

The quality of each trial will be categorized into “low bias,” “unclear bias,” and “high bias” according to the following items: adequacy of generation of the allocation sequence, allocation concealment, blinding of participants and personal, blinding of outcome assessors, incomplete outcome data, selected reporting the results and other sources of bias (such as comparable baseline characteristic, inclusion and exclusion criteria).

#### 2.4.4 Assessment of reporting biases

Reporting biases and small-study effects will be detected by funnel plot and Egger test if there are 10 more studies included in this Meta-analysis. For Egger test, *p*-value of <.10 was considered to indicate the existence of reporting biases and small study effects.

#### 2.4.5 Data analysis

We will use Review Manager software version 5.3 provided by the Cochrane Collaboration for the data analyze and synthesis. Binary outcomes will be summarized using risk ratio with 95% confidence interval (CI) for relative effect. Continuous outcomes will be summarized by using weighted mean difference with 95% confidence interval. We will use random-effect model for meta-analysis in this review according to research recommendations.^[26]^

Statistical heterogeneity will be assessed by *X*^2^ and *I*^2^ statistical tests. Where *P*-value ≥ .1 and *I*^2^ ≤ 50%, there is no obvious statistical heterogeneity among the studies, and then we will choose fixed effect model (FEM) to synthesize the data. On the contrary, if *P* value < .1 or *I*^2^ > 50%, it indicates that there is a considerable heterogeneity, we will integrate data by the random effect model (REM). Meta-analysis will be performed when the statistical heterogeneity is acceptable (*P*-value ≥ .1 and *I*^2^ ≤ 50%), otherwise, subgroup analysis will be applied to explore the influence of potential factors on the outcome measures. We will conduct subgroup analyses by different race, age, gender, course of treatment, and different type of GHHD (intervention forms, pharmaceutical dosage form, dosage, etc). We will conduct sensitivity analyses by omitting studies 1 by 1 to probe the impact of an individual study. If a meta-analysis cannot be performed, we will conduct descriptive analysis instead.

#### 2.4.6 Patient and public involvement

This is a meta-analysis study based on previously published data, so patient and public involvement will not be included in this study.

#### 2.4.7 Ethics and dissemination

Ethical approval will not be required as this is a protocol for systematic review and meta-analysis. The findings of this study will be disseminated to a peer-reviewed journal and presented at a relevant conference.

#### 2.4.8 Evidence assessed

The quality of evidence for this study will be assessed by “Grades of Recommendations Assessment, Development, and Evaluation (GRADE)” standard established by the World Health Organization and international organizations.^[27]^ To achieve transparency and simplification, the quality of evidence is divided into 4 levels in GRADE system: high, medium, low, and very low. We will employ GRADE profiler 3.2 for analysis.

## 3 Discussion

Rotavirus enteritis in children, according to its clinical manifestations, should belong to the category of “diarrhea” in TCM. According to the 2016 Clinical Practice Guidelines for Children with Acute Infectious Diarrhea in China, the survey results show that there will be 2 epidemic trends each year, 1 concentrated in June to August, the main pathogens are diarrheal Escherichia coli and Shigella, and the other is concentrated in October to December, the main pathogen is rotavirus.^[21]^

According to the statistics of the World Health Organization in recent years, 130 million infants and children under age of 5 with diarrhea caused by group A rotavirus infection every year in the world. Including 873,000 deaths. Since rotavirus invades the intestinal villi epithelial cells and the virus replicates in the intestinal villi epithelial cells, the absorption of sodium, glucose and water is reduced, and the activity of digestive enzymes in the intestinal tract is also reduced, all of these leading to osmotic diarrhea and secretory diarrhea caused by activation of NSP4 and the intestinal nervous system.^[28,29]^ Traditional Chinese Medicine has a long history in treating diarrhea. GHHD is 1 of the oldest classic formula of TCM that applied in the field of diarrhea treatment. In recent years, lots of pharmacological evidences have shown that GHHD could stop diarrhea and vomiting. In this study, we would like to provides a comprehensive assessment to whether GHHD is beneficial for children under 5 rotavirus enteritis. And we attempted to provide high-quality evidence for the clinical efficacy and safety of GHHD. Moreover, RCTs will be included in our studies and appear to be high quality and low risk of bias. However, there may be some deficiencies in this systematic review. First of all, the TCM clinical trial design that we intend to include is difficult to have double-blind method throughout the whole research. Second, the variety of race, age, gender, intervention forms, pharmaceutical dosage form, dosage, and treatment course may result in higher clinical and statistical heterogeneity. We hope that this study result will give some new insight into this field, which, to some extent, can help physicians and patients.

## Author contributions

**Conceptualization**: Yifang Wu, Xiang Tu.

**Data curation**: Yifang Wu, Xiao Liang, Jing Chen, Tianhong Zhang.

**Formal analysis**: Xiao Liang, Jing Chen.

**Methodology**: Yifang Wu, Jingrong Jiang, Xiao Liang, Xuemei Wan.

**Project administration**: Sen Zhong.

**Resources**: Yifang Wu, Xiang Tu, Sen Zhong.

**Software**: Yifang Wu, Xiang Tu, Sen Zhong.

**Supervision**: Xiang Tu, Sen Zhong.

**Writing – original draft**: Yifang Wu.

**Writing – review & editing**: Sen Zhong.
